# Lapatinib and poziotinib overcome ABCB1-mediated paclitaxel resistance in ovarian cancer

**DOI:** 10.1371/journal.pone.0254205

**Published:** 2021-08-04

**Authors:** J. Robert McCorkle, Justin W. Gorski, Jinpeng Liu, McKayla B. Riggs, Anthony B. McDowell, Nan Lin, Chi Wang, Frederick R. Ueland, Jill M. Kolesar

**Affiliations:** 1 Markey Cancer Center, College of Medicine, University of Kentucky, Lexington, KY, United States of America; 2 Department of Obstetrics and Gynecology, Division of Gynecologic Oncology, College of Medicine, University of Kentucky, Lexington, KY, United States of America; 3 College of Pharmacy, University of Kentucky, Lexington, KY, United States of America; 4 Department of Biostatistics, College of Public Health, University of Kentucky, Lexington, KY, United States of America; Columbia University, UNITED STATES

## Abstract

Conventional frontline treatment for ovarian cancer consists of successive chemotherapy cycles of paclitaxel and platinum. Despite the initial favorable responses for most patients, chemotherapy resistance frequently leads to recurrent or refractory disease. New treatment strategies that circumvent or prevent mechanisms of resistance are needed to improve ovarian cancer therapy. We established *in vitro* paclitaxel-resistant ovarian cancer cell line and organoid models. Gene expression differences in resistant and sensitive lines were analyzed by RNA sequencing. We manipulated candidate genes associated with paclitaxel resistance using siRNA or small molecule inhibitors, and then screened the cells for paclitaxel sensitivity using cell viability assays. We used the Bliss independence model to evaluate the anti-proliferative synergy for drug combinations. ABCB1 expression was upregulated in paclitaxel-resistant TOV-21G (q < 1x10^-300^), OVCAR3 (q = 7.4x10^-156^) and novel ovarian tumor organoid (p = 2.4x10^-4^) models. Previous reports have shown some tyrosine kinase inhibitors can inhibit ABCB1 function. We tested a panel of tyrosine kinase inhibitors for the ability to sensitize resistant ABCB1-overexpressing ovarian cancer cell lines to paclitaxel. We observed synergy when we combined poziotinib or lapatinib with paclitaxel in resistant TOV-21G and OVCAR3 cells. Silencing ABCB1 expression in paclitaxel-resistant TOV-21G and OVCAR3 cells reduced paclitaxel IC_50_ by 20.7 and 6.2-fold, respectively. Furthermore, we demonstrated direct inhibition of paclitaxel-induced ABCB1 transporter activity by both lapatinib and poziotinib. In conclusion, lapatinib and poziotinib combined with paclitaxel synergizes to inhibit the proliferation of ABCB1-overexpressing ovarian cancer cells *in vitro*. The addition of FDA-approved lapatinib to second-line paclitaxel therapy is a promising strategy for patients with recurrent ovarian cancer.

## Introduction

Ovarian cancer is a devastating disease that affects 1 in 70 women during their lifetime. There will be an estimated 22,000 new cases of ovarian cancer in 2020, and approximately 14,000 women in the United States will die from the disease [1]. The five-year overall survival rate remains less than 50%, making ovarian cancer the most deadly gynecologic malignancy [2]. More than two-thirds of patients are diagnosed at an advanced stage with five-year survival rates of 36% and 17% for stage III and IV disease, respectively [2]. The recommended treatment of women with advanced-stage ovarian cancer includes platinum-based doublet chemotherapy [3, 4]. Unfortunately, over 80% of patients with advanced ovarian cancer who achieve a complete response with conventional first-line chemotherapy will develop recurrent disease [5]. Despite modest response rates for all regimens (20–30%), dose-dense paclitaxel is a preferred agent in treating platinum-resistant recurrence [6–8]. Virtually all relapsed patients will die of disease, highlighting the pressing need for novel therapies.

Chemotherapy resistance is categorized as either inherent or acquired. Inherent resistance exists before chemotherapy exposure, while acquired resistance develops in response to chemotherapy administration. Mechanisms of acquired resistance to paclitaxel include alterations in microtubules, dysregulation of apoptosis, and increased cellular drug efflux [9]. Multidrug resistance (MDR) is a pervasive impediment to the successful treatment of solid tumors, including ovarian cancer. The MDR mechanism includes elevated expression of the ATP-binding cassette (ABC) family of transmembrane transporters that can reduce intracellular drug levels and limit therapeutic efficacy. ATP-binding cassette subfamily B, member 1 (ABCB1), also known as P-glycoprotein (P-gp) or multidrug resistance protein 1 (MDR1), was the first ABC transporter identified and is the best characterized ABC family member [10–12]. ABCB1 functions to protect cells from the damage of xenobiotic and toxic substances, including chemotherapeutic agents (e.g., taxanes, vinca alkaloids, anthracyclines) [13]. We observe higher paclitaxel IC_50_ (i.e., the concentration required to inhibit proliferation in 50% of cells) in immortalized human ovarian cancer cell lines and patient-derived organoids with elevated ABCB1 expression. Similar observations have been reported across a wide variety of cancer cell lines [14–16]. Elevated ABCB1 expression and poor paclitaxel response has also been associated with unfavorable clinical outcomes for ovarian cancer patients [17].

Clinical development of small molecule inhibitors of ABC transporters to reverse MDR has been ongoing for more than three decades. Initially, ABCB1 inhibitors were used for non-oncologic indications (e.g., verapamil, quinine, and cyclosporine), but the high doses necessary to inhibit ABCB1 function proved to be too toxic [18, 19]. Better tolerated second- and third-generation ABCB1 inhibitors demonstrate a more favorable therapeutic window, though new clinical applications remain elusive [20–22]. Similarly, many tyrosine kinase inhibitors (TKIs) (e.g., dasatinib, vandetanib, lapatinib), can inhibit ABCB1 function, albeit from off-target activity [23–29]. Recent *in vitro* studies have shown lapatinib can partially reverse multidrug resistance in ABCB1-overexpressing ovarian cancer cells, findings further supported through *in vivo* mouse models [30].

In the current study, we aimed to better understand acquired paclitaxel resistance in ovarian cancer and identify small molecules that possess anti-cancer synergy when combined with paclitaxel in resistant ovarian cancer. A variety of ovarian cancer models were employed, including immortalized cell lines and primary tumor organoids established from several different histotypes, in hopes of finding a treatment strategy with broad applicability to gynecological cancers. We report that among a panel of tested TKIs, lapatinib, and poziotinib demonstrated the strongest synergy in combination with paclitaxel in ABCB1-overexpressing human ovarian cancer cells. Others have previously reported synergistic antitumor activity using paclitaxel and lapatinib in combination for esophageal and ovarian cancer [31, 32]. To our knowledge, this is the first report of anticancer synergy using poziotinib in combination with paclitaxel in ovarian cancer, although poziotinib-mediated inhibition of ABCB1 activity was recently reported in colon cancer cells [33]. Both poziotinib and lapatinib are TKIs that primarily target ErbB protein tyrosine kinases EGFR, ERBB2, and ERBB4 [34–36]. Evidence suggests lapatinib inhibits ABCB1 activity, but the mechanism remains unclear [24]. We show that its synergistic anti-cancer effect, when combined with paclitaxel, is independent of EGFR, ERBB2, or ERBB4 antagonism in human ovarian cancer cell models. Moreover, poziotinib and lapatinib appear to inhibit ABCB1 via direct interaction with the transporter. We also observe elevated ABCB1 expression associated with paclitaxel resistance in primary ovarian tumor organoids underscoring potential clinical relevance. These findings encourage further investigation of whether lapatinib can be combined with paclitaxel to improve the chemotherapeutic efficacy and survival in ovarian cancer patients.

## Materials and methods

### Drug-resistant cell lines

We purchased ovarian cancer cell lines TOV-21G [37] (ATCC^®^ CRL-11730^™^) and NIH:OVCAR-3 (OVCAR3) [38] (ATCC^®^ HTB-161^™^) directly from ATCC and absence of mycoplasma was confirmed independently. TOV-21G cells were maintained subconfluent at 37°C, 5% CO_2_ in 1:1 (v:v) mixture of Medium 199:MCDB 105 supplemented with 15% fetal bovine serum (FBS). OVCAR3 cells were grown as subconfluent monolayers in high glucose (4500 mg/L) RPMI-1640 with 0.01 mg/mL bovine insulin and 20% FBS at 37°C, 5% CO_2_. Paclitaxel-resistant cells were established from parental lines by incubating cells in paclitaxel-containing growth media using recurrent 48-hour treatment cycles until a stable pool of resistant clones was confirmed. Clinically relevant paclitaxel concentrations of 25, 50 and 150 nM were used for the first cycle and the highest concentration that allowed for outgrowth of viable cells was selected for subsequent cycles. The concentration used and recovery time needed for cells to repopulate between cycles was determined independently for each cell line. We treated TOV-21G cells with 3 cycles of 150 nM paclitaxel for 48 hours each, every 2 weeks. We treated OVCAR-3 cells with 5 cycles of 25 nM paclitaxel for 48 hours each, every 2 to 3 weeks. The stability of the resistant phenotype was verified by routine testing with dose-response proliferation assays, for a minimum of 8 passages following the final treatment cycle.

### Organoids

Ovarian tumor tissue was obtained at the time of debulking surgery from patients who provided written, informed consent to the use of their tissue for establishment of tumor organoids and cell lines, as approved by the University of Kentucky Institutional Review Board. Establishment of organoids and drug sensitivity testing was conducted by the organoid modeling laboratory at Tempus (Chicago, IL). Tissue was enzymatically dissociated and *de novo* organoids were established in Matrigel® Growth Factor Reduced Basement Membrane Matrix (Corning) *in vitro* using factor defined media [39] and grown at 37°C, 5% CO_2_. Representative micrographs were H&E stained and compared with the primary tumor to confirm concordant histology. We also performed mutational concordance analysis by comparison of sequencing results between the primary tumor specimen and the resultant tumor organoids. For cytotoxicity assays, organoids were enzymatically dissociated into single cells and seeded in individual wells of 384-well plates. Organoids were cultured under normal conditions for 72 hours before administering paclitaxel treatment (1000 nM, 100 nM, 10 nM, 1 nM, 0.01 nM) in quadruplicate wells per dose. Experiments were terminated after treating cells for 72 hours. Cell viability was measured using CellTiter 96 Aqueous One Solution Cell Proliferation assay (Promega) to determine EC_50_ for each sample.

### RNA sequencing

Total RNA was extracted from ovarian cancer cell lines with RNeasy Plus Universal Mini Kit (Qiagen) followed by whole transcriptome sequencing. RNA from each cell line was analyzed in triplicate. RNA quality and quantity were assessed with an Agilent Bioanalyzer 2100 RNA Nano chip. TruSeq Stranded Total RNA Prep Kit (Illumina) was used to generate ribosomal RNA-depleted libraries which were sequenced as 100 base pair, single-end reads on an Illumina HiSeq 2500 in rapid mode by the Markey Cancer Center’s Oncogenomics Shared Resource Facility at the University of Kentucky. An average depth of 4.9x10^7^ single-end reads per sample was achieved. RNA from tumor organoids were sequenced by Tempus (Chicago, IL) using the proprietary xT sequencing platform with an exome capture-based RNA-seq methodology [40]. Sequencing reads were trimmed and filtered to remove adapter sequences and low-quality reads using Trimmomatic (V0.39) [41]. Read alignments were mapped to Ensembl GRCh38 transcripts annotation (release 82), using STAR aligner in the RSEM software [42]. On average, there were 4.2x10^7^ (~84.1%) uniquely mapped reads per sample and 3.8x10^7^ (~75.6%) reads mapped to exons in the ovarian cancer cell lines. Depth of coverage summary is provided in S8 Fig. We used R (version 3.5.0) and the Bioconductor (version 3.10) package *edgeR* for normalization and differential expression analysis [43]. The raw counts of the samples in comparison were first normalized within samples using read counts aligning to each gene per million mapped reads (CPM). We excluded from analysis genes that were unexpressed or lowly expressed (no sample with CPM > 1). The read counts were further normalized between samples using TMM (**T**rimmed **M**eans of **M** values) to account for the library size variance. Within *edgeR*, the read counts were fit to a negative binomial distribution model to estimate variance, and differential expression was subsequently analyzed using the “exactTest” function [43]. Significant differentially expressed genes (control vs. paclitaxel-resistant) had log_2_ fold change ≥ 1 or ≤ -1 and q-value < 0.05. RNA-seq data are available at the Gene Expression Omnibus (GEO) under accession number GSE172016.

### Proliferation assays

Cells were seeded in white-walled 96-well microplates at 3x10^3^ cells per well in 100 μL growth media and incubated for 24 hours at 37°C, 5% CO_2_ to allow cells to attach. Subsequently, the growth media was removed and replaced with fresh media containing serially diluted drug(s) of interest or blank media for untreated controls. Within an experiment, each drug concentration was tested in duplicate. For paclitaxel, drug concentrations ranged from 3000 nM to 0.017 nM. Lapatinib concentrations spanned from 50 μM to 0.08 μM. After treatment, we incubated cells an additional 96 hours, then determined cell viability of drug-treated cells relative to untreated control cells (% viability) using the CellTiter-Glo 2.0 viability assay (Promega). Luminescence was measured using a Varioskan LUX multimode microplate reader (ThermoFisher Scientific). Dose-response curves were then fit to the data (four parameter log-logistic model), and we calculated IC_50_ values with R statistical software (version 3.5.0), package *drc* (version 3.0) [44]. Each drug was analyzed with each cell line using data from a minimum of three independent experiments.

### Drug combination/synergy analysis

We produced dose-response matrix data using cell proliferation assays as described above. We tested pairs of drugs alone and in combination with each of five serially diluted concentrations and cell viability was measured using CellTiter-Glo 2.0 (Promega). For every drug pair, each concentration was tested in combination in at least 3 independent experiments in a 6 x 6 matrix design. Percent viability for each combination was determined by dividing drug-treated signals by untreated signals and multiplying by 100. Paclitaxel (1000, 167, 28, 4.6, 0.77, 0 nM) was tested in combination with lapatinib (50000, 10000, 2000, 400, 80, 0 nM), poziotinib (12500, 4167, 1389, 463, 154, 0 nM), vandetanib (10000, 2000, 400, 80, 16, 0 nM), dacomitinib (10000, 3333, 1111, 370, 123, 0 nM), AZ5104 (5000, 500, 50, 5, 0.5, 0 nM) and allitinib (12500, 1250, 125, 12.5, 1.25, 0 nM). The percentage of viable cells relative to untreated control cells was used to assess synergy for each drug combination using the *synergyfinder* package (version 1.10) within R (version 3.5.0) [45]. We used the Bliss independence model [46] for synergy scoring.

### siRNA transfection

We used siGENOME SMARTpool siRNA reagents (Dharmacon) for the RNAi-mediated knockdown of human EGFR, ERBB2, ERBB4, and ABCB1. Ovarian cancer cell lines were transiently transfected with 15 nM siRNA, including the siGENOME Non-Targeting siRNA Pool #2, using DharmaFECT1 transfection reagent (Dharmacon) according to the manufacturer’s instructions. Cells were harvested 24 hours after transfection and either seeded in 96-well microplates for cell proliferation assays or re-plated for expansion prior to RNA extraction.

### Real-time PCR

RNA was extracted from ovarian cancer cell lines with RNeasy Plus Universal Mini Kit (Qiagen) and 1 μg of each sample was converted to cDNA using High-Capacity cDNA Reverse Transcription Kit (ThermoFisher Scientific) with random primers and MultiScribe Reverse Transcriptase. Reverse transcription reactions were performed in a VeritiPro Thermal Cycler (Applied Biosystems) under the following conditions: 25°C for 10 minutes, 37°C for 120 minutes, 85°C for 5 minutes. Real-time semi-quantitative PCR to measure gene expression was employed using FAM-MGB labeled TaqMan Gene Expression Assays (ThermoFisher Scientific). TaqMan Advanced Master Mix (ThermoFisher Scientific) was used to assess expression of human ABCB1 (ATP binding cassette subfamily B member 1; assay ID Hs00184500_m1), EGFR (epidermal growth factor receptor; assay ID Hs01076090_m1), ERBB2 (erb-b2 receptor tyrosine kinase 2; assay ID Hs01001580_m1), ERBB4 (erb-b2 receptor tyrosine kinase 4; assay ID Hs00955522_m1), relative to MRPL19 (mitochondrial ribosomal protein L19; assay ID Hs00608519_m1) in triplicate. The reaction mixture consisted of 1X Master Mix, 1X TaqMan Assay and 1 μL template cDNA in 20 μL final volume. PCR was carried out using a QuantStudio 3 Real-Time PCR instrument in “fast” run mode with the following conditions for all genes: 2 minute hold at 50°C, 2 minute hold at 95°C, followed by 40 cycles of amplification for 1 second at 95°C and 20 seconds at 60°C. Relative expression was evaluated across samples in QuantStudio Software (Applied Biosystems) using the Comparative C_T_ (ΔΔC_T_) method.

### ABCB1 activity

Transporter activity of ABCB1 was assessed in the presence of lapatinib, poziotinib, paclitaxel and verapamil (positive control) using the Pgp-Glo Assay System with P-glycoprotein (Promega) according the manufacturer’s instructions [47]. Briefly, single agent 10 μM lapatinib, 10 μM poziotinib, 0.5 μM paclitaxel, 200 μM verapamil, or 10 μM lapatinib or poziotinib combined with 0.5 μM paclitaxel or 100 μM verapamil, were incubated with 0.5 mg/mL P-gp membranes (supplied with kit) in 5 mM MgATP for 60 minutes on a heating block set to 37°C. Reactions were stopped by the addition of 50 μL ATP Detection Reagent. Plates were mixed on an orbital shaker for 10 seconds at 500 RPM then incubated 20 minutes at room temperature protected from light. Luminescence was then measured in quadruplicate wells for each sample with a 1 second exposure using a Varioskan LUX Multimode microplate reader (ThermoFisher Scientific). Luminescent signals (RLU) from three independent experiments were analyzed. RLU values from untreated (baseline), verapamil-treated (positive control) and experimental samples were subtracted from sodium vanadate-treated samples (negative control) to determine change in luminescence. These values were normalized to baseline levels to determine relative change in ABCB1 activity.

### Statistical analysis

Statistical analyses were performed using R (version 3.6.3) or GraphPad Prism (version 5.01). For RNAseq analyses, differential expression was analyzed using the “exactTest” function [43]. Significant differentially expressed genes (control vs. paclitaxel-resistant) had log_2_ fold change ≥ 1 or ≤ -1 and q-value < 0.05 (q < 0.1 for tumor organoids). Synergy studies were conducted in quadruplicate and Bliss scores were compared (control vs. PacR) using unpaired two-tailed t-tests. Real-time PCR data was analyzed by comparing ΔC_T_ values using unpaired two-tailed t-tests (2 samples) or 1-way ANOVA followed by Tukey’s Multiple Comparison Tests (time course). Data was collected in triplicate across at least three independent experiments. Results are presented as mean ± standard error of the mean. Pairwise comparisons of IC_50_ values were assessed using two-tailed unpaired t-tests were used to assess differences between means of a minimum of three independent experiments. IC_50_ values were calculated by fitting the experimental data to four-parameter log-logistic curves using package *drc* (version 3.0) in R. Median P-gp activity determined from quadruplicate measurements across three independent experiments was analyzed using Kruskal-Wallis test followed by pairwise comparisons using Conover-Iman tests.

## Results

**Fig 1:**
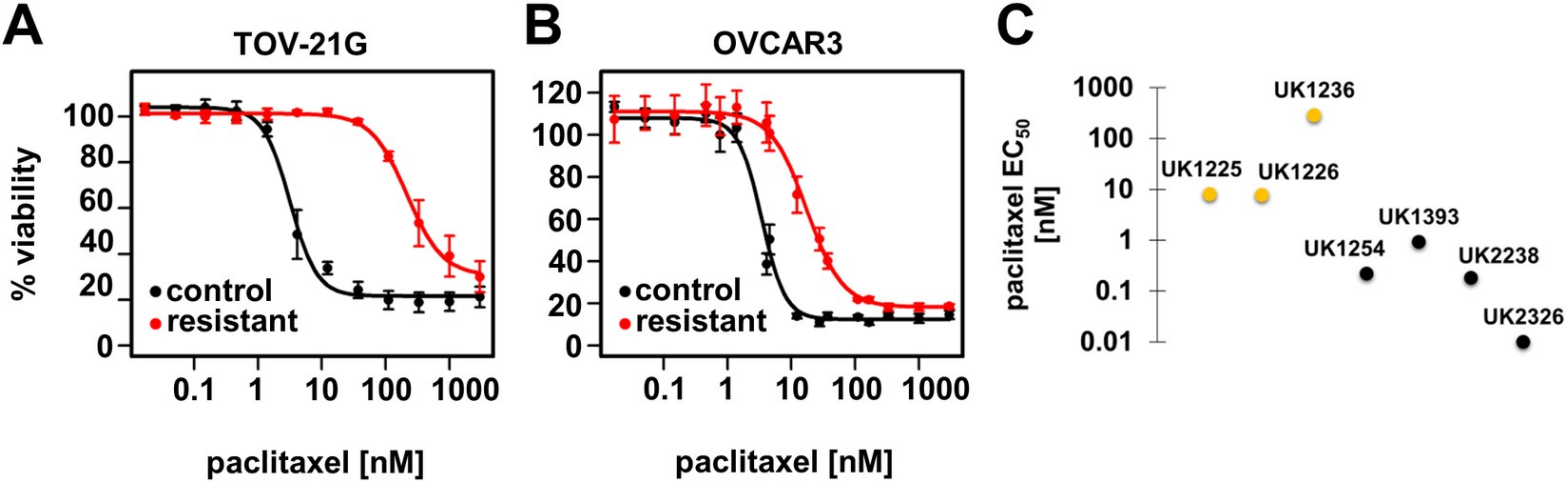
Ovarian cancer cell lines exhibit varied responses to paclitaxel treatment. Paclitaxel-resistant (PacR) and parental control ovarian cancer cell lines, TOV-21G and OVCAR3, were treated with serially diluted doses of paclitaxel for 96 hours *in vitro*. Cell viability is displayed at each concentration tested relative to untreated cells for the control (black) and PacR (red) cell lines of (A) TOV-21G and (B) OVCAR3 cells. Dose response curves were fit to the data and IC_50_ values were calculated using four-parameter log-logistic models. (C) Ovarian tumor organoid cell lines’ paclitaxel EC_50_ values. Resistant lines are shown in gold and sensitive lines are shown in black.

We used primary organoid and immortalized cell line models to study the genetic basis of paclitaxel resistance in ovarian cancer. We generated models of acquired paclitaxel resistance from the human clear cell ovarian carcinoma cell line, TOV-21G, and the human papillary serous ovarian carcinoma line, OVCAR3. Cell lines were grown in monolayers and treated with clinically-achievable concentrations of paclitaxel (25–150 nM) in 48-hour increments then allowed to recover in drug-free culture media. Upon repopulation, cells were treated with additional cycles of paclitaxel until we established stable pools of resistant cells. Resistant cell lines exhibited increases in paclitaxel IC_50_ ranging from 6.5-fold for OVCAR3 (26.6 nM in resistant versus 4.1 nM in control) to 94-fold for TOV-21G (403.1 nM in resistant versus 4.3 nM in control) (Fig 1A and 1B).

In parallel, primary tumor organoid cell lines were established from seven unique ovarian cancer patients using tissue acquired from primary debulking surgeries (Table 1) [48]. Most organoid lines were developed from high grade serous carcinoma specimens (n = 5) isolated from the ovary (n = 2), fallopian tube (n = 2) or omentum (n = 1). We derived the remaining two organoid lines from low-grade adenocarcinomas, one serous, and one endometrioid. Paclitaxel resistance was analyzed in these novel organoid cell lines, with EC_50_ values spanning more than four orders of magnitude (0.01–285 nM), with a median EC_50_ of 0.92 nM. We designated the four organoid lines with EC_50_ values less than or equal to the median EC_50_ as “sensitive” to paclitaxel and three lines with EC_50_ values greater than the median as “resistant” (Fig 1C). We established five lines from chemotherapy naïve specimens, and two from patients who received neoadjuvant chemotherapy consisting of carboplatin and paclitaxel. Of the neoadjuvant treated organoid lines, one was resistant and had the highest paclitaxel EC_50_ (UK1236), while the other was sensitive to paclitaxel (UK1254).

**Table 1:** Clinical information for organoid tissue donors.

Organoid ID	Age at diagnosis (years)	Race	Histotype	Stage	Grade	Tissue^1^	BRCA status
UK1225	62	White	endometrioid adenocarcinoma	IA	1 (well differentiated)	Ovary	WT
UK1226	57	White	serous adenocarcinoma with marked psammoma bodies	IIIA2	1 (low grade)	Ovary	WT
UK1236	47	White	residual serous adenocarcinoma	IVA	3	Ovary	WT
UK1254	49	White	residual serous carcinoma	IVA	3	Ovary	WT
UK1393	47	White	serous carcinoma	IIIC	3	Omentum	WT
UK2238	58	White	serous carcinoma	IIIA2	3	Fallopian tube	BRCA1 c.2071delA (germline)
UK2326	63	White	serous carcinoma	IIIC	3	Fallopian tube	WT

^1^ Site of origin

**Fig 2:**
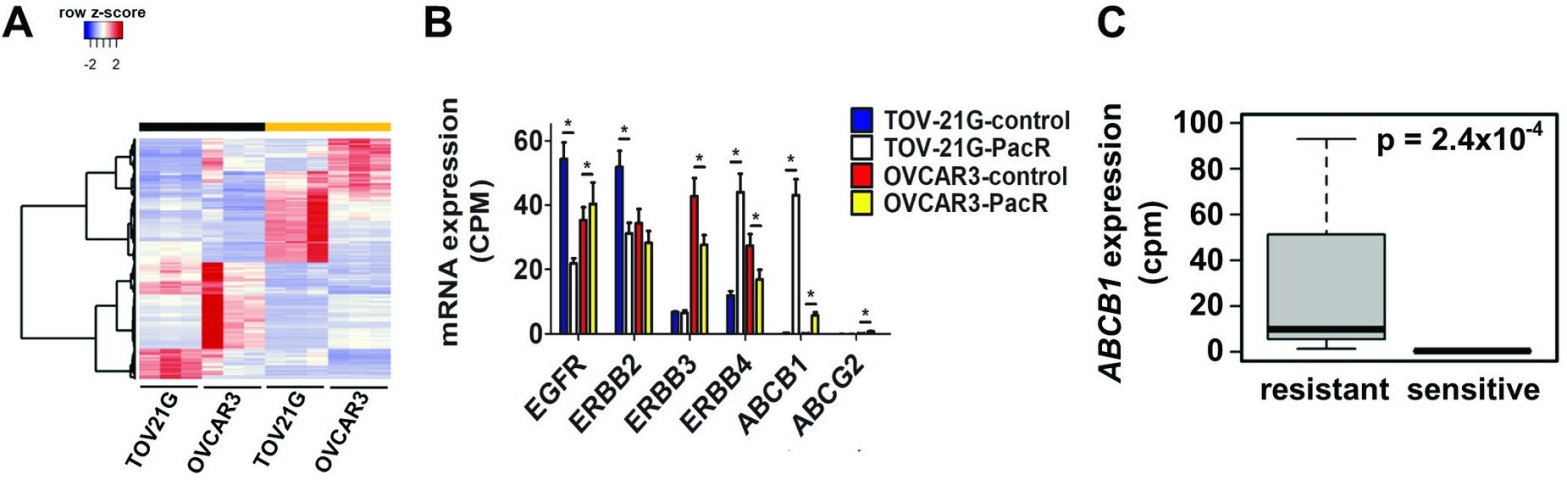
ABCB1 overexpression among recurrent gene expression differences tracking with paclitaxel resistance in ovarian cancer. (A) Supervised hierarchical clustering of 229 common transcripts phenotypically segregated 111 down-regulated and 118 up-regulated transcripts in PacR cells (orange bar) from control cells (black bar). (B) mRNA expression levels measured by RNAseq in control and PacR cells were compared within cell lines for *EGFR*, *ERBB2*, *ERBB3*, *ERBB4*, *ABCB1* and *ABCG2* (* q < 0.05). (C) *ABCB1* mRNA expression levels measured by RNAseq compared in paclitaxel-sensitive and -resistant ovarian cancer organoids. Statistical significance determined by exact test (*edgeR*).

To better understand the molecular basis for the paclitaxel-resistance (PacR) phenotype, gene expression profiling was conducted on the PacR ovarian cancer cell lines and matched parental controls. Gene expression differences associated with paclitaxel resistance revealed common changes in certain transcripts irrespective of the cell line. There were 1795 differentially expressed transcripts identified in the TOV-21G-PacR cells compared to control (FDR q < 0.05; log_2_ fold change ≥ 1 or ≤ -1; “TOV21G” in S1 Table). For OVCAR3, we found 2407 differentially expressed transcripts (“OVCAR3” in S1 Table). A group of 229 genes showed similar expression patterns across the PacR cell lines, 118 of which exhibited increased expression; 111 genes had reduced expression (Fig 2A; “Common” in S1 Table). ABCB1 expression was strongly induced in PacR cells and was the most statistically significant of the common differentially expressed genes (Fig 2B; q < 1.0x10^-300^ and q = 7.4x10^-156^ in TOV-21G and OVCAR3 cells, respectively). Similarly, we analyzed gene expression differences among sensitive and resistant ovarian cancer organoid lines. As seen in the immortalized cell lines, elevated ABCB1 expression was associated with paclitaxel resistance in the organoid lines, too (Fig 2C; p = 2.4x10^-4^). Further examination of ABCB1 splice variants, gene fusions and mutations in our models detected no alterations. A synonymous polymorphism (rs2214102; chr7:87600185 T>C) was found in TOV-21G cells.

**Fig 3:**
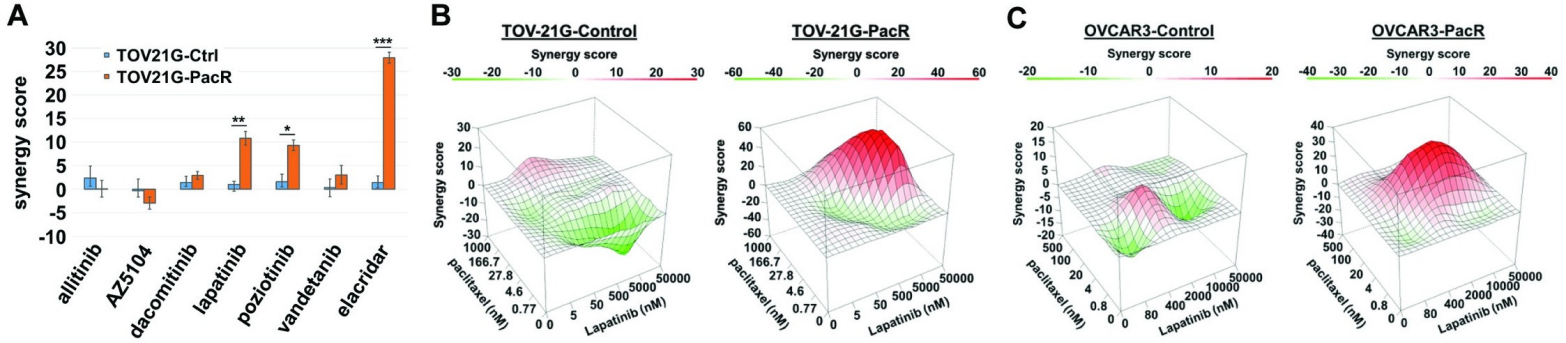
Lapatinib and poziotinib demonstrate anti-proliferative synergy when combined with paclitaxel. (A) A panel of TKIs were screened for anti-proliferative synergy when combined with paclitaxel on TOV-21G-control and–PacR derivative cell lines. The ABCB1-specific inhibitor, elacridar, was included as a positive control. Average Bliss synergy scores across drug combinations (minimum of 3 independent experiments) are summarized as averages +/- standard error of the mean. Unpaired two-tailed t-tests were performed for each drug (control vs. PacR; * p < 0.05; ** p < 0.005; *** p < 0.0005). (B) Representative surface response model plots of synergy analyses using a Bliss independence model for paclitaxel in combination with lapatinib are depicted for control and PacR TOV-21G and (C) OVCAR3 cell lines.

We sought to identify compounds that showed anti-proliferative synergy when combined with paclitaxel in resistant P-gp-overexpressing ovarian cancer cells. Evidence has shown some TKIs can inhibit P-gp activity [23–29]; therefore, we used a panel of investigational and clinically approved TKIs to screen for synergy. We focused on inhibitors of the ERBB gene family due to expression differences in resistant ovarian cancer cells associated with the resistant phenotype (Fig 2B). Since ERBB4 was differentially expressed in resistant cells of both lines, we chose ERBB family TKIs active against ERBB4 for further investigation. For the initial screen, we treated parental and PacR TOV-21G cells with poziotinib, dacomitinib, AZ-5104, allitinib (AST-1306), vandetanib, or lapatinib alone and in combination with paclitaxel, and then assayed for cell viability. We calculated the average synergy scores based upon the Bliss independence model for each combination tested [45, 46] (Fig 3A). The ABCB1-specific inhibitor, elacridar, was included as a positive control and showed the strongest synergy with paclitaxel, with a mean Bliss score of 1.5 versus 27.9 for control and PacR cells, respectively (p = 1.3x10^-4^). Anti-proliferative synergy was also seen when paclitaxel was combined with lapatinib (p = 0.0030) or poziotinib (p = 0.015). These findings were then validated in OVCAR3 cells for lapatinib (p = 0.025) and poziotinib (p = 0.039) (S1 Fig). Representative surface response models show similar Bliss synergy scores over the range of concentrations tested for the lapatinib plus paclitaxel combination in TOV-21G and OVCAR3-PacR cells, with no synergy in control cells with low basal expression of ABCB1 (Fig 3B and 3C).

**Fig 4:**
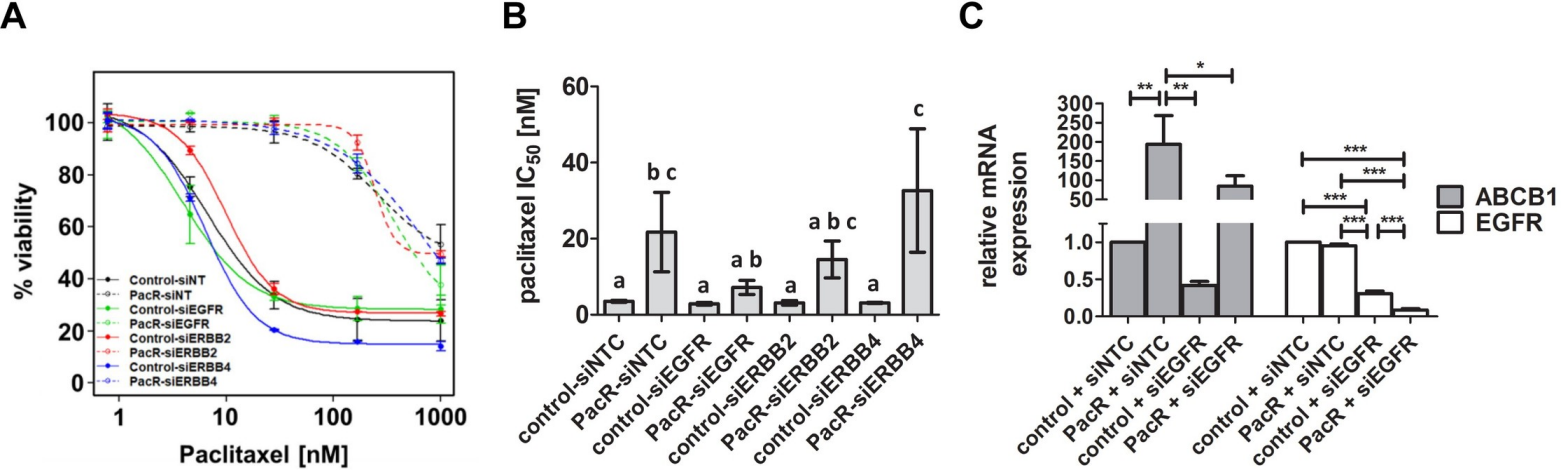
Differential regulation of ABCB1 expression by EGFR in ovarian cancer cell lines. (A) Dose response curves following siRNA knockdown of EGFR, ERBB2 or ERBB4 in TOV-21G-control and–PacR cells indicating cell viability after 96 hours of exposure to paclitaxel. (B) Paclitaxel IC50 in OVCAR3-control and -PacR cells following knock-down of EGFR, ERBB2, and ERBB4 expression. Bar plots depict average IC50 +/- standard error of the mean (SEM). ANOVA p < 0.0001. Bars not sharing subscripts are significantly different (Tukey’s p < 0.05). (C) ABCB1 and EGFR expression measured with real-time PCR 72 hours after siEGFR transfection in OVCAR3 cells. Relative expression was compared using one-way ANOVA (ABCB1 p = 0.001; EGFR p < 0.0001) and Tukey’s Multiple Comparison Tests (* p < 0.05, ** p < 0.01, *** p < 0.001).

Lapatinib and poziotinib inhibit the ErbB family of tyrosine kinases. To assess the potential contribution of ErbB receptors on paclitaxel resistance in ovarian cancer, silencing RNAs (siRNA) were used to knockdown expression of EGFR, ERBB2, and ERBB4 in the control and PacR cells (S1 and S3 Figs). Following siRNA transfection, cells were treated with lapatinib and paclitaxel alone and in combination for 96 hours. Sustained down regulation of gene expression was confirmed at 120 hours post-transfection by real-time PCR (S4 and S5 Figs). Cells were inherently resistant to lapatinib, and we observed no differences among control and PacR cells (S6 Fig). Blocking the expression of EGFR, ERBB2, or ERBB4 did not alter paclitaxel-induced cytotoxicity in the TOV-21G cell line when compared to cells that received non-target siRNA (Fig 4A). However, we observed a significant decrease in paclitaxel IC_50_ in OVCAR3-PacR cells treated with EGFR siRNA (Fig 4B). We then measured ABCB1 expression following knock-down of EGFR in OVCAR3 cells and found significantly lower ABCB1 mRNA levels in siEGFR-treated cells compared to the non-target (siNTC) control (Fig 4C). A similar siEGFR-induced reduction in ABCB1 expression was not observed in the TOV21G cells (S7 Fig).

**Fig 5:**
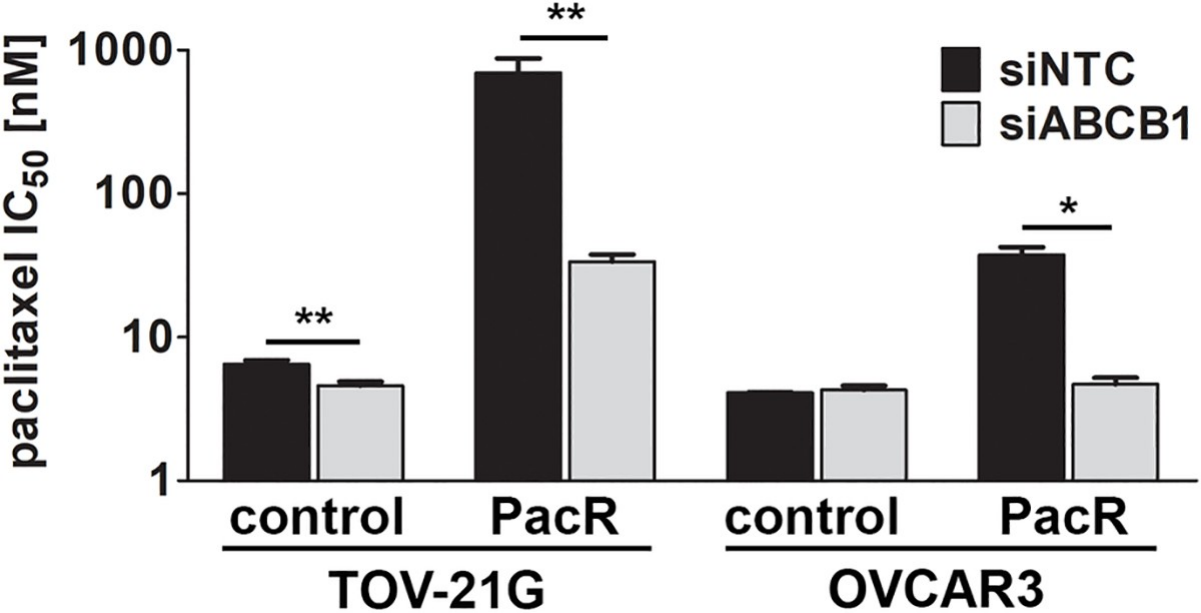
ABCB1 overexpression is sufficient for paclitaxel resistance in ovarian cancer. Cell viability following paclitaxel treatment was examined after silencing ABCB1 expression in TOV-21G and OVCAR3 cells. IC_50_ values were compared across non-target siRNA (siNTC) and ABCB1 siRNA (siABCB1) transfected cells using unpaired two-tailed t-tests. * p < 0.05; ** p < 0.005.

In addition to ErbB receptors, lapatinib reportedly inhibits the function of at least two ATP-binding cassette (ABC) transporters, ABCB1 and ABCG2 [24]. These transporters promote cytotoxic resistance by enhancing cellular efflux of chemotherapeutic drugs, including paclitaxel. Expression levels of ABCG2 are low or undetectable in our cell lines (Fig 2B); thus, any contribution to the resistant phenotype is negligible in these models. ABCB1 expression was reduced by siRNA in TOV-21G and OVCAR3-control and PacR cells. We then measured paclitaxel-induced cytotoxicity. Silencing ABCB1 in TOV-21G-PacR cells significantly reduced paclitaxel IC_50_ 21.6-fold when compared to cells receiving non-target siRNA (p < 0.005; Fig 5). In comparison, silencing ABCB1 in the TOV-21G-control cells had a more modest effect on paclitaxel sensitivity; IC_50_ was 6.8 nM among cells receiving non-target siRNA versus 4.9 nM for cells receiving ABCB1 siRNA (p < 0.005). Similarly, in OVCAR3-control cells, there was no difference in IC_50_ after silencing ABCB1 expression. We did observe a significant reduction in IC_50_ in the OVCAR3-PacR cells with silenced ABCB1 expression (p < 0.05; Fig 5).

**Fig 6:**
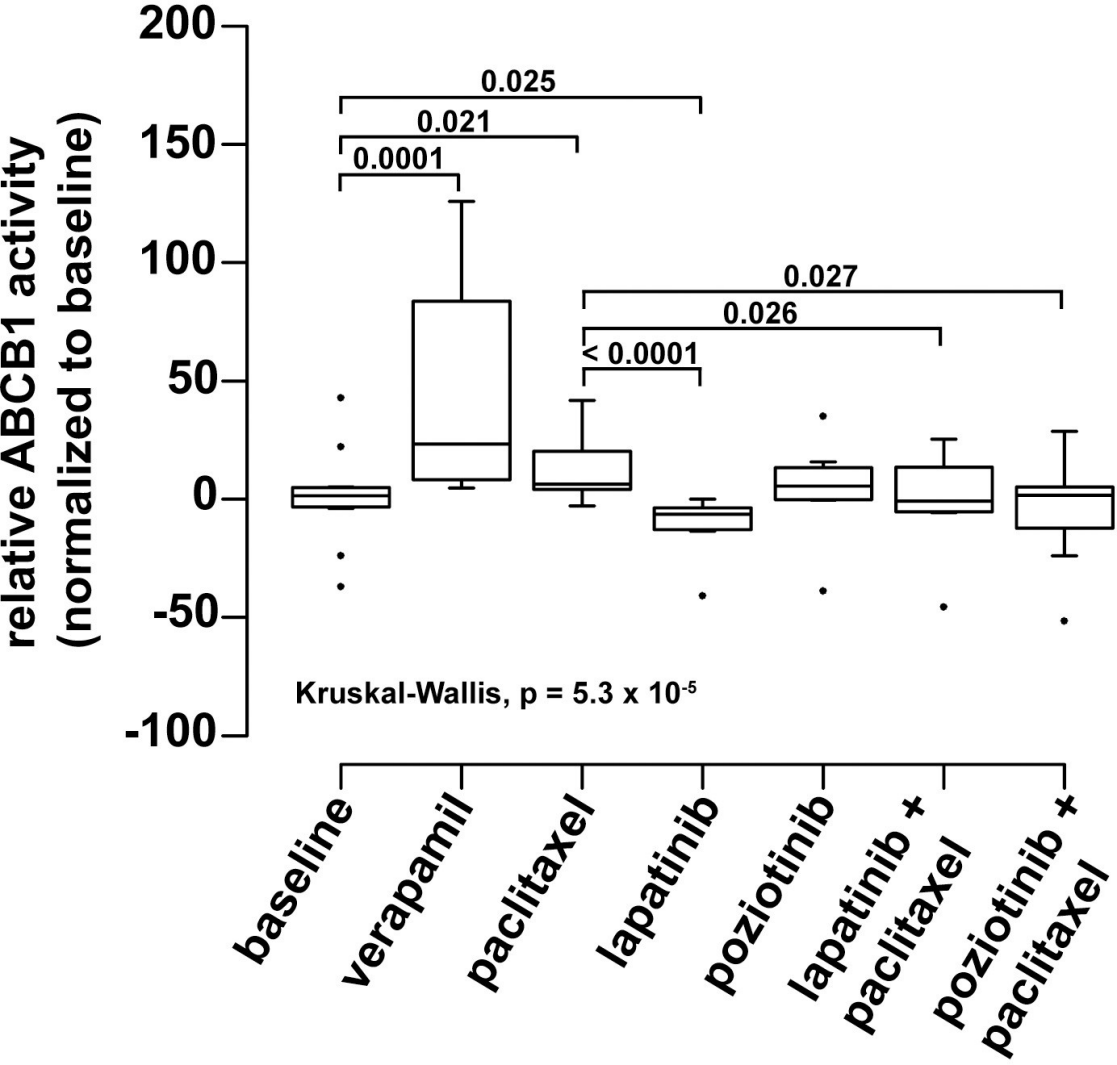
Lapatinib and poziotinib directly inhibit ABCB1-mediated paclitaxel transport. ABCB1 ATPase activity was measured in untreated (baseline) and drug-treated recombinant human ABCB1 membranes and normalized to baseline activity. Verapamil served as positive control. Box and whisker plots depict the median activity, interquartile range (boxes) with whiskers extending to data points up to 1.5x the interquartile range (IQR). Outlier values greater than 1.5x IQR are shown as closed circles. Statistical significance was determined using Kruskal-Wallis test followed by Conover-Iman tests for pair-wise comparisons.

To investigate the mechanism of lapatinib and poziotinib-mediated ABCB1 inhibition, we examined their effect on transporter activity. ABCB1 is an ATP-dependent transporter and ABCB1 substrates will stimulate ATPase activity. ABCB1 ATPase activity was measured using the Pgp-Glo Assay System (Promega) [47] in the presence of verapamil (positive control), paclitaxel, lapatinib and poziotinib, and with paclitaxel in combination with lapatinib and poziotinib. As shown in Fig 6, single agent verapamil and paclitaxel stimulated ABCB1 ATPase activity 23-fold (p = 0.0001) and 6-fold (p = 0.021), respectively, compared to baseline ABCB1 activity (no added substrate). As single agents, lapatinib inhibited ABCB1 activity approximately 6-fold (p = 0.025) compared to baseline while poziotinib showed no significant difference. Combining lapatinib or poziotinib with paclitaxel significantly reduced paclitaxel-induced ABCB1 activity (p = 0.026 and p = 0.027, respectively; Fig 6).

## Discussion

Resistance to chemotherapy profoundly impacts cancer outcomes by limiting the clinical benefit of cancer treatment. Many mechanisms of resistance have been described, with the upregulation of drug efflux transporters (e.g., ABCB1) being one of the more commonly observed *in vitro* [13]. The current study demonstrates that ABCB1 upregulation is the major driver of paclitaxel resistance in two different human ovarian cancer cell line models of acquired resistance. Silencing ABCB1 expression with sequence-specific siRNAs significantly reduced paclitaxel IC_50_ in resistant cells (Fig 5). Resistance was completely overcome in OVCAR3-PacR cells; however, sensitivity was only partially restored in TOV-21G-PacR cells. This is most likely due to the highly elevated ABCB1 expression in TOV-21G-PacR cells (Fig 2B) combined with the incomplete silencing of ABCB1 expression in these cells (S4A Fig). While expression was significantly reduced in the TOV-21G-PacR-siABCB1 versus–siNTC cells, ABCB1 expression remained elevated relative to the TOV-21G-control cell lines. Although OVCAR3-PacR-siABCB1 cells similarly maintained elevated ABCB1 expression compared to OVCAR3-control cells (S5A Fig), we did not observe increased paclitaxel IC_50_. The magnitude of ABCB1 upregulation is much lower in OVCAR3-PacR cells compared to TOV-21G-PacR cells (Fig 2B). It is possible that the ABCB1 siRNAs sufficiently reduce ABCB1 expression in OVCAR3-PacR cells below a threshold required for paclitaxel resistance that could not be achieved in the TOV-21G-PacR cells. An alternative technology that could provide a more complete knock-out of *ABCB1* (e.g., CRISPR) may be necessary to completely overcome resistance in the TOV-21G-PacR cells.

We show that for ovarian cancer cells overexpressing ABCB1, the combination treatment of lapatinib and paclitaxel has synergistic effects, sensitizing otherwise resistant cells to paclitaxel. This finding is in agreement with other studies in a variety of cancer cell types [24, 25, 31, 49]. We have also shown for the first time in ovarian cancer cells that poziotinib, currently in late phase clinical trials for patients with NSCLC and an *ERBB2* exon 20 insertion mutation [50], has a similar effect when combined with paclitaxel in ABCB1-overexpressing ovarian cancer cells. However, poziotinib requires higher concentrations to achieve comparable synergy to lapatinib with paclitaxel. Poziotinib and lapatinib are inhibitors of ErbB receptors, therefore, we aimed to determine if expression of ErbB receptors influenced paclitaxel sensitivity. Silencing expression of EGFR, ERBB2 or ERBB4 had no effect on paclitaxel-mediated cytotoxicity in TOV-21G-control or TOV-21G-PacR cells (Fig 4A). However, silencing EGFR significantly reduced paclitaxel IC_50_ in OVCAR3-PacR cells (Fig 4B). We found that when EGFR expression was reduced in OVCAR3-PacR cells, a concomitant decrease in ABCB1 expression occurred (Fig 4C), leading to lower paclitaxel IC_50_ in these cells. Interestingly, silencing EGFR in the TOV-21G cells did not alter ABCB1 expression (S7 Fig) highlighting cell line-specific differences in how ABCB1 expression is controlled. Regulation of ABCB1 expression by EGFR has been previously reported [51–53] and it would appear EGFR is, at least in part, mediating ABCB1 overexpression in the OVCAR3-PacR cells.

Other TKIs targeting the ErbB receptor family, specifically allitinib, dacomitinib, vandetanib and AZ5104, did not sensitize cells to paclitaxel treatment in our model despite previous reports of ABCB1 inhibition by dacomitinib in colon cancer cell lines (Fig 3A) [54]. While variability exists in the ability of different TKIs to inhibit ABCB1 function, these data support continued clinical investigation of the combination of paclitaxel and lapatinib.

As shown in Fig 6, the ability of lapatinib and poziotinib to reduce paclitaxel efficacy is likely due to direct inhibition of ABCB1 function. Based on the observed stimulation of ABCB1 ATPase activity, paclitaxel is a substrate of ABCB1. The reduction in paclitaxel-induced ATPase activity *in vitro* when lapatinib or poziotinib are present demonstrates physical interference with ABCB1 function. Our results are in agreement with others that have proposed a mechanism whereby TKIs, including lapatinib and poziotinib, physically bind to and inhibit ABCB1 rendering the transporter non-functional [27, 33].

Demonstration of clinical relevance for ABCB1 upregulation on chemoresistance in ovarian cancer has been inconsistent; however, recent reports highlight the importance of ABCB1-overexpression in the treatment of recurrent ovarian cancer [55, 56]. This is not unexpected since patients with recurrent ovarian cancer have already received paclitaxel, an ABCB1 substrate, as part of their primary therapy [57]. Neoadjuvant chemotherapy (carboplatin/paclitaxel doublet) is also commonly prescribed before primary debulking surgery. Thus, primary cancer tissue samples obtained after neoadjuvant chemotherapy may commonly express efflux transporters. We developed our ovarian tumor organoid models from seven primary ovarian cancer patients, two of whom received neoadjuvant chemotherapy. Of these two, one had the highest level of ABCB1 expression (UK1236), ~10-fold higher than the next highest expresser, and the other had undetectable ABCB1 expression (UK1254). It is unclear whether epigenetic or other molecular mechanisms underlie differences observed in these models for induction of ABCB1 expression following neoadjuvant chemotherapy. Regardless, our new ovarian cancer organoid cell lines are providing a clinically relevant validation of the link between ABCB1 upregulation and paclitaxel resistance in ovarian cancer, consistent with previously established immortalized human cell lines in the laboratory setting.

The combination of lapatinib and paclitaxel has been previously used for the treatment of advanced solid malignancies [58–63], but exclusively in the context of ERBB2-overexpression. While results were inconsistent, most of these trials showed this combination produced a favorable overall response rate in ERBB2-overexpressing tumors and had manageable toxicities. Re-evaluation of this combination therapy is warranted, with a new focus on ABCB1-overexpressing ovarian cancers. This treatment strategy is attractive for several reasons. First, dose-dense paclitaxel is a preferred second-line therapy for resistant or refractory ovarian cancers. Since lapatinib is already FDA-approved, its addition to paclitaxel as a second-line therapy can move quickly into early phase trials. Second, the pharmacology of lapatinib is well understood and has a known toxicity profile. Using lapatinib with paclitaxel could allow for dose-reductions of paclitaxel that may lower the incidence of dose-limiting toxicities associated with paclitaxel use (e.g., neuropathy, bone marrow suppression). Lastly, lapatinib may be available soon as a generic version of Tykerb (Novartis) which the FDA granted approval for in September 2020 [50]. This should substantially reduce the cost of lapatinib and make it a more affordable treatment option.

From genome-wide screens, we show ABCB1 overexpression consistently associated with paclitaxel resistance across various human ovarian cancer cell line models. While immortalized cell lines are considered poor clinical surrogates due to their relative homogeneity, we identified elevated ABCB1 expression in clinically relevant, paclitaxel-resistant tumor organoid models, as well. Limitations of this work include small sample size, so it will be important to show similar associations across a broader collection of clinical samples. Although lapatinib shows the highest degree of synergy with paclitaxel in our study, the possibility that other untested agents are more effective than lapatinib in ABCB1-overexpressing ovarian cancers cannot be ruled out.

There is a pressing need for new therapeutic options for ovarian cancer and our findings identify a promising approach for patients with relapsed ovarian cancer. We show that ABCB1-overexpressing ovarian cancers are particularly sensitive to paclitaxel combined with lapatinib in the laboratory setting and that ABCB1 overexpression is common and predictive of paclitaxel sensitivity in patient-derived ovarian organoids. The clinical investigation of this combination is relatively low risk with high reward potential. Repurposing lapatinib to enhance paclitaxel efficacy represents an opportune therapeutic strategy to improve outcomes for relapsed/refractory ovarian cancer patients.

## Supporting information

S1 Table Differentially expressed genes associated with paclitaxel resistance in ovarian cancer cell lines. (XLSX)

S1 Fig Drug synergy in OVCAR3 cells. Average Bliss synergy scores across drug combinations (paclitaxel + lapatinib or paclitaxel + poziotinib) from 4 independent experiments of OVCAR3-control and -PacR cells are summarized as averages +/- standard error of the mean. The ABCB1 inhibitor, elacridar, serves as positive control. Unpaired two-tailed t-tests were performed for each drug (control vs. PacR; * p < 0.05; ** p < 0.01).
(TIF)

S2 Fig Real-time PCR analysis of siRNA efficacy in TOV-21G cells. Relative expression of *ABCB1*, *EGFR*, *ERBB2*, and *ERBB4* following siRNA transfection in TOV-21G cells. Significant differences in expression (ΔC_T_) were determined using unpaired two-tailed t-tests (* p < 0.001).
(TIF)

S3 Fig Real-time PCR analysis of siRNA efficacy in OVCAR3 cells. Relative expression of *ABCB1*, *EGFR*, *ERBB2*, and *ERBB4* following siRNA transfection in OVCAR3 cells. Significant differences in expression (ΔC_T_) were determined using unpaired two-tailed t-tests (* p < 0.001).
(TIF)

S4 Fig Time course real-time PCR analysis of siRNA efficacy in TOV-21G-control and -PacR cells. Gene expression of *ABCB1*, *EGFR*, *ERBB2* and *ERBB4* were measured in TOV-21G control and paclitaxel-resistant cells at 48 hours and 120 hours post-transfection. Expression was analyzed relative to control cells at time 0 using *MRPL19* as the endogenous reference gene. Error bars indicate 95% confidence intervals for relative expression from triplicate measurements. Samples not sharing subscripts are significantly different (p < 0.05).
(TIF)

S5 Fig Time course real-time PCR analysis of siRNA efficacy in OVCAR3-control and -PacR cells. Gene expression of *ABCB1*, *EGFR*, *ERBB2* and *ERBB4* were measured in OVCAR3-control and paclitaxel-resistant cells at 48 hours and 120 hours post-transfection. Expression was analyzed relative to control cells at time 0 using *MRPL19* as the endogenous reference gene. Error bars indicate 95% confidence intervals for relative expression from triplicate measurements. Samples not sharing subscripts are significantly different (p < 0.05).
(TIF)

S6 Fig Lapatinib sensitivity in control and paclitaxel-resistant TOV-21G and OVCAR3 cells. Dose response assays were used to determine relative *in vitro* cytotoxicity after 96 hours of exposure to lapatinib in resistant and control ovarian cancer cell lines, TOV-21G and OVCAR3.
(TIF)

S7 Fig ABCB1 expression in TOV-21G-control and–PacR cells following ErbB family gene silencing. Real-time PCR analysis of ABCB1 expression 48 hours post-transfection of siEGFR, siERBB2, siERBB4 or siNTC control siRNA constructs. Bar plots depict expression relative to TOV-21G-control-siNTC cells with error bars depicting 95% confidence intervals. MRPL19 expression was used as the calibrator. One-way ANOVA (p < 0.0001) and Tukey’s Multiple Comparison tests were used to determine statistical significance. Bars not sharing a common subscript are significantly different (Tukey’s p < 0.05).
(TIF)

S8 Fig Read depth for RNA sequencing analysis of immortalized cell lines. For each immortalized ovarian cancer cell line analyzed by RNA-seq, depth of coverage (x-axis) is plotted against the proportion of genes greater than or equal to a given read depth (y-axis).
(TIF)
