# Supplementary figures and images for: Lapatinib and poziotinib overcome ABCB1-mediated paclitaxel resistance in ovarian cancer
Source: PLoS One. 2021 Aug 4;16(8):e0254205. doi: 10.1371/journal.pone.0254205 (PMC8336885; doi:10.1371/journal.pone.0254205)

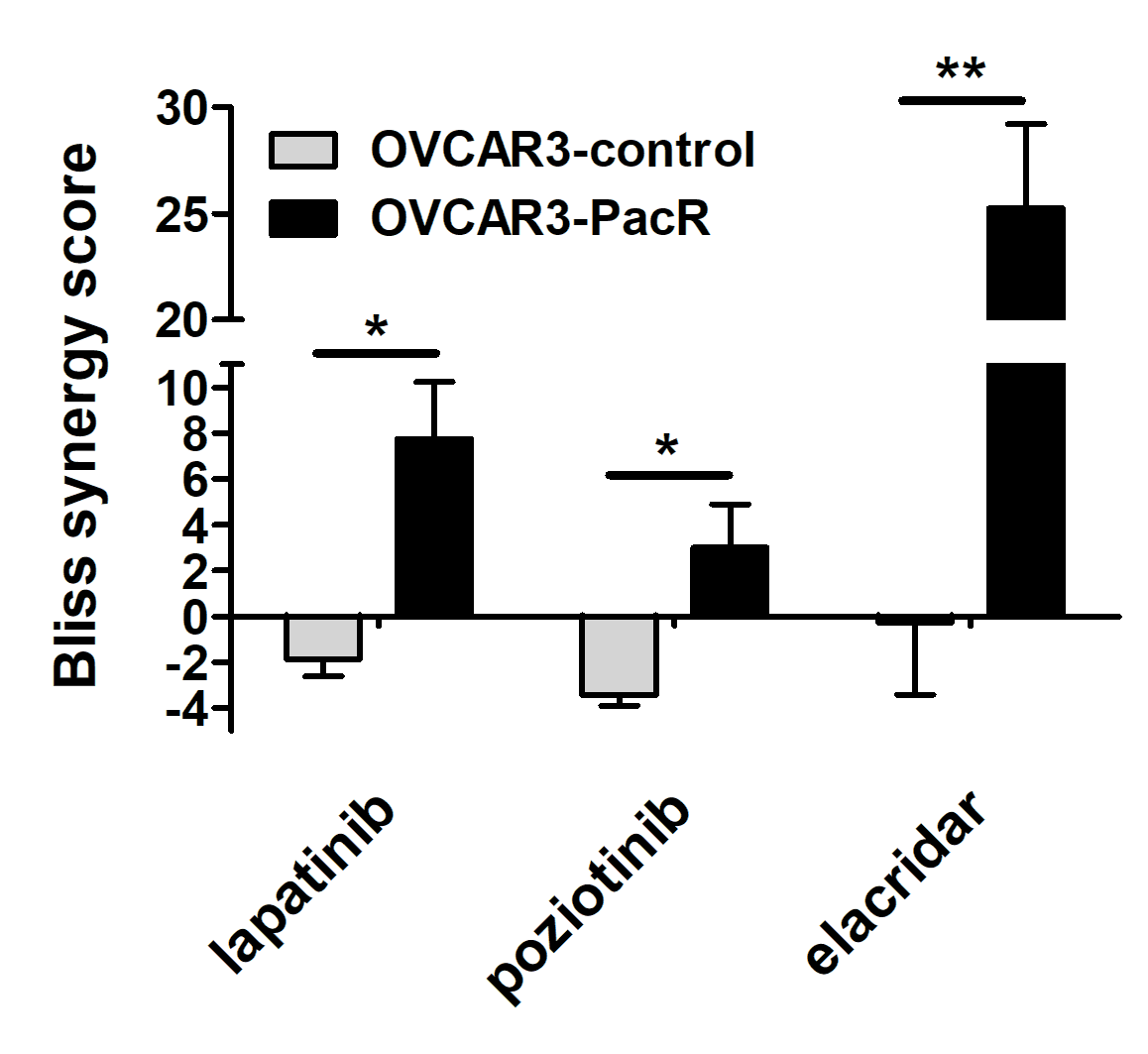

Supplement: S1 Fig — Average Bliss synergy scores across drug combinations (paclitaxel + lapatinib or paclitaxel + poziotinib) from 4 independent experiments of OVCAR3-control and -PacR cells are summarized as averages +/- standard error of the mean. The ABCB1 inhibitor, elacridar, serves as positive control. Unpaired two-tailed t-tests were performed for each drug (control vs. PacR; * p < 0.05; ** p < 0.01). (TIF) [file pone.0254205.s002.tif]

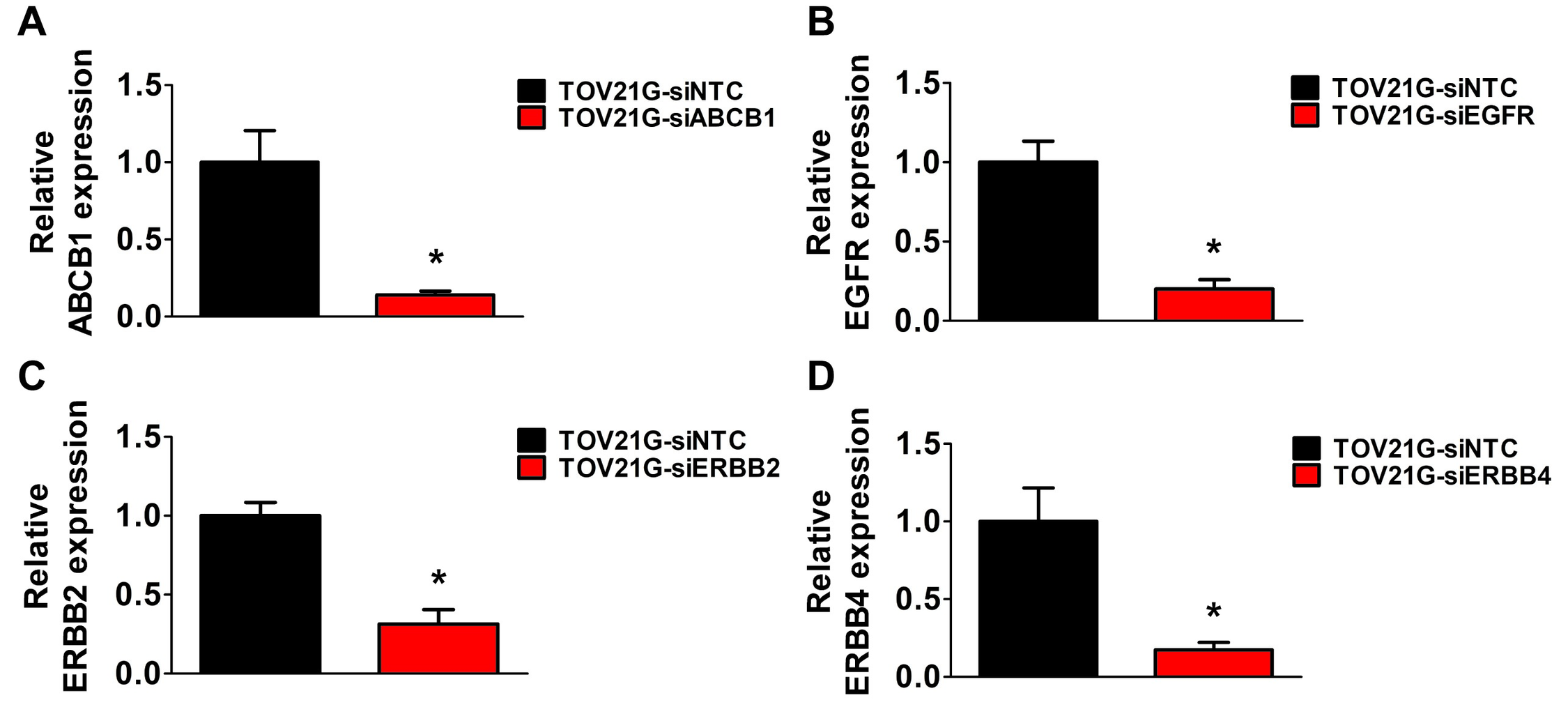

Supplement: S2 Fig — Relative expression of ABCB1, EGFR, ERBB2, and ERBB4 following siRNA transfection in TOV-21G cells. Significant differences in expression (ΔCT) were determined using unpaired two-tailed t-tests (* p < 0.001). (TIF) [file pone.0254205.s003.tif]

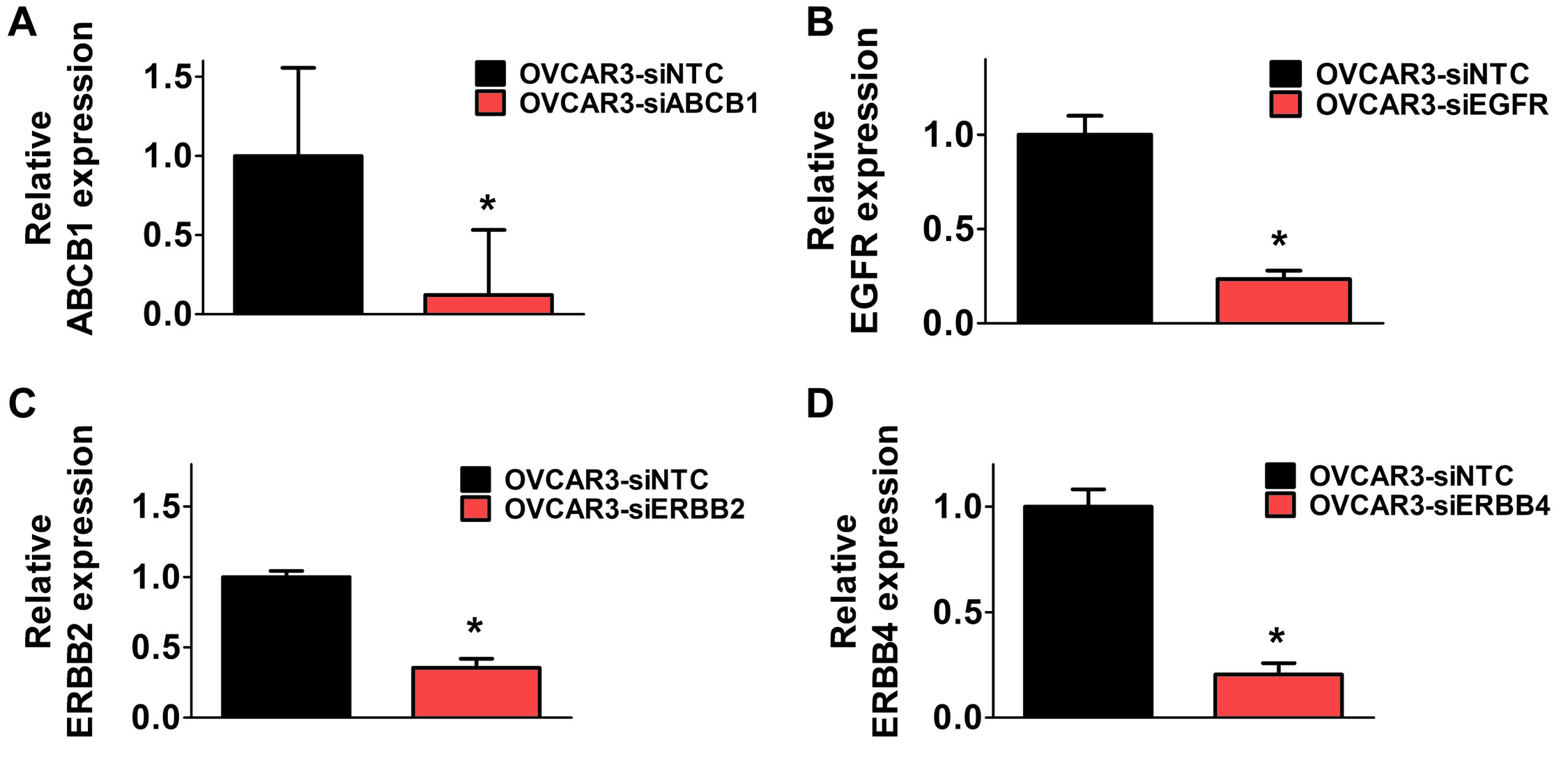

Supplement: S3 Fig — Relative expression of ABCB1, EGFR, ERBB2, and ERBB4 following siRNA transfection in OVCAR3 cells. Significant differences in expression (ΔCT) were determined using unpaired two-tailed t-tests (* p < 0.001). (TIF) [file pone.0254205.s004.tif]

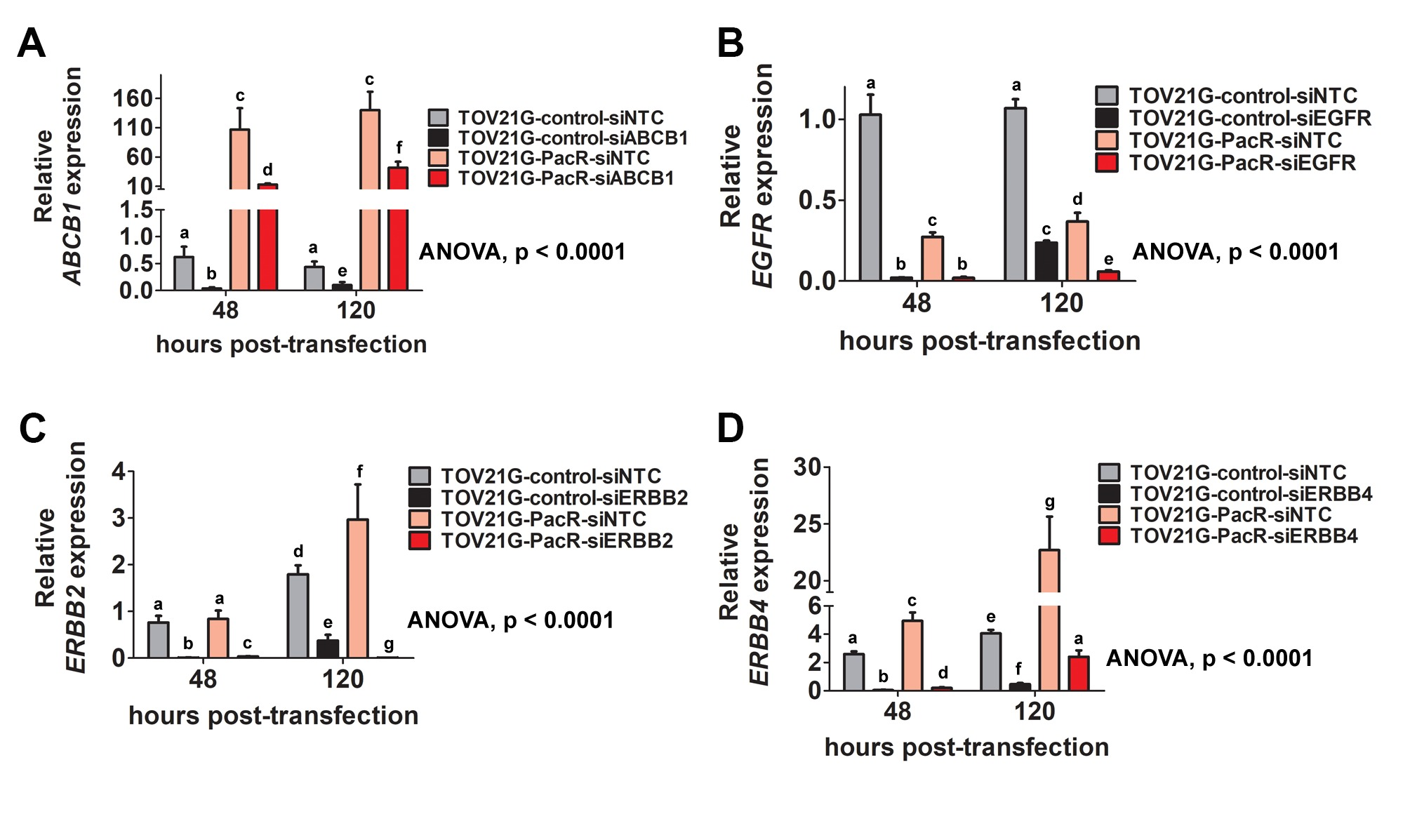

Supplement: S4 Fig — Gene expression of ABCB1, EGFR, ERBB2 and ERBB4 were measured in TOV-21G control and paclitaxel-resistant cells at 48 hours and 120 hours post-transfection. Expression was analyzed relative to control cells at time 0 using MRPL19 as the endogenous reference gene. Error bars indicate 95% confidence intervals for relative expression from triplicate measurements. Samples not sharing subscripts are significantly different (p < 0.05). (TIF) [file pone.0254205.s005.tif]

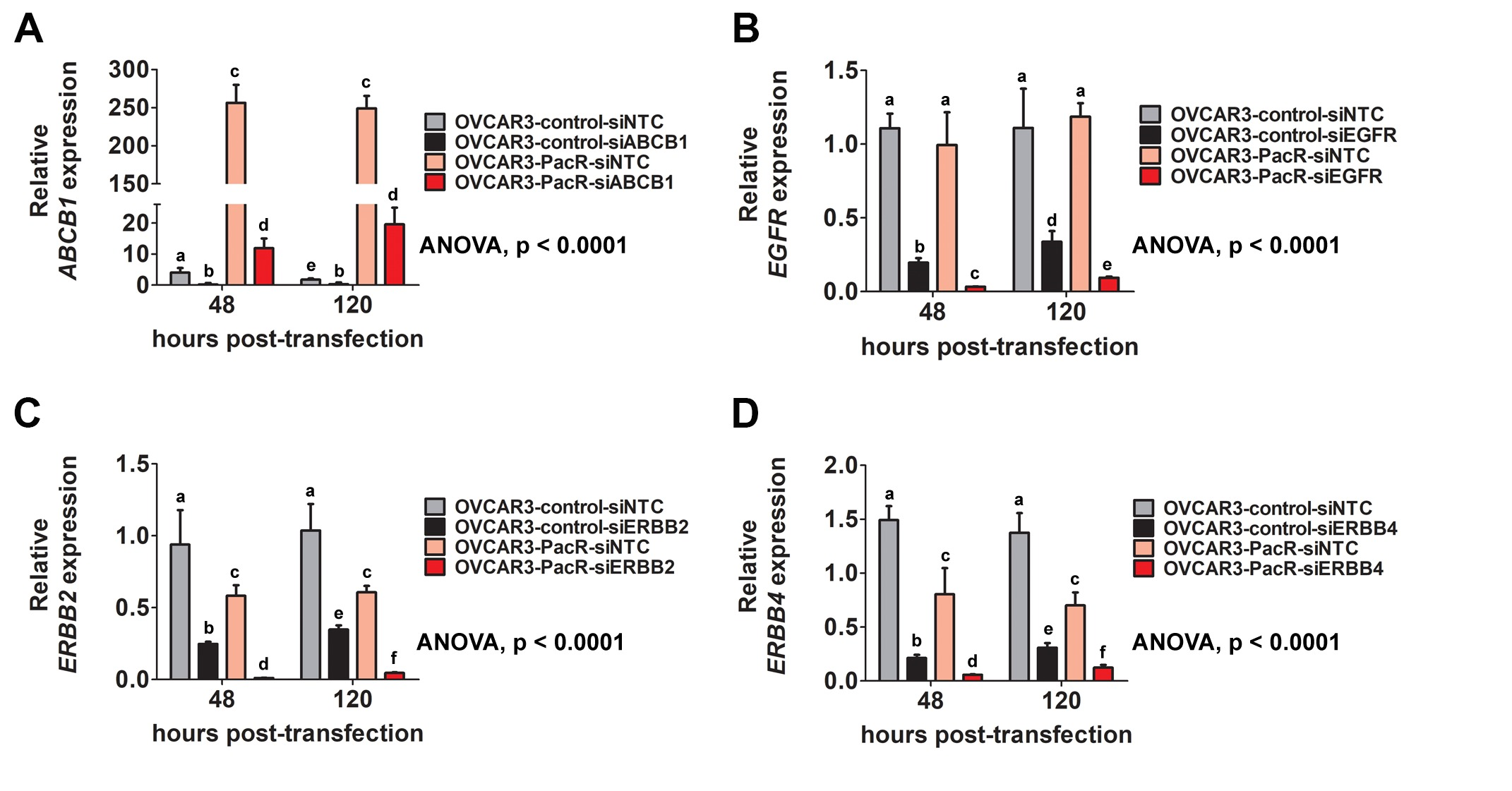

Supplement: S5 Fig — Gene expression of ABCB1, EGFR, ERBB2 and ERBB4 were measured in OVCAR3-control and paclitaxel-resistant cells at 48 hours and 120 hours post-transfection. Expression was analyzed relative to control cells at time 0 using MRPL19 as the endogenous reference gene. Error bars indicate 95% confidence intervals for relative expression from triplicate measurements. Samples not sharing subscripts are significantly different (p < 0.05). (TIF) [file pone.0254205.s006.tif]

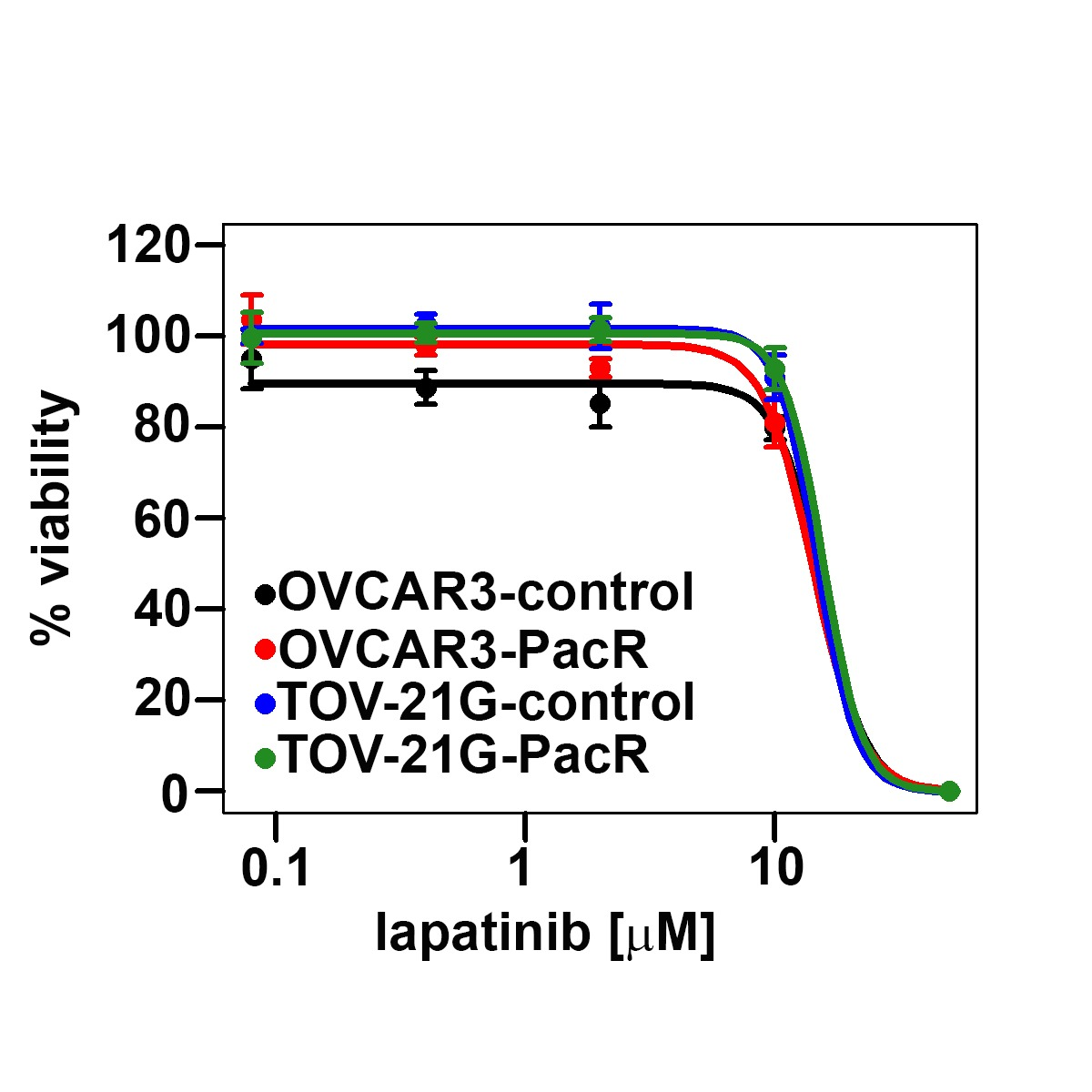

Supplement: S6 Fig — Dose response assays were used to determine relative in vitro cytotoxicity after 96 hours of exposure to lapatinib in resistant and control ovarian cancer cell lines, TOV-21G and OVCAR3. (TIF) [file pone.0254205.s007.tif]

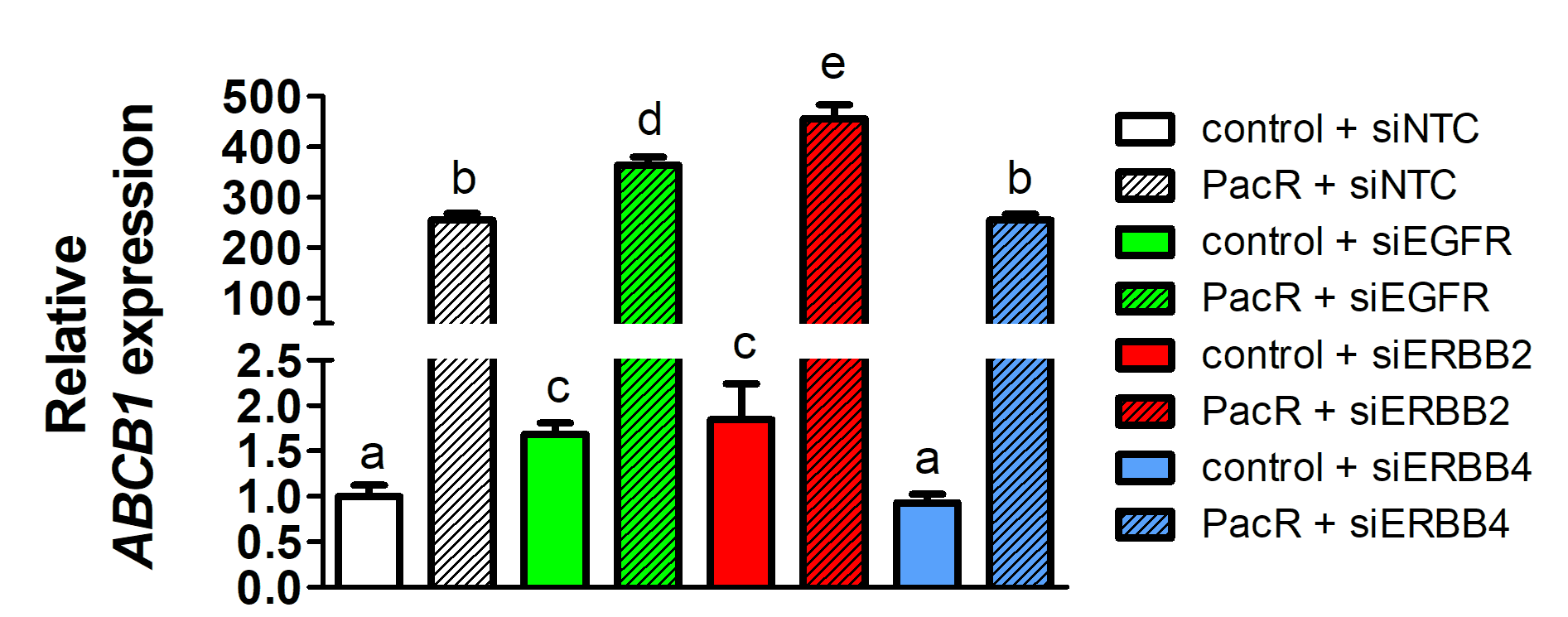

Supplement: S7 Fig — Real-time PCR analysis of ABCB1 expression 48 hours post-transfection of siEGFR, siERBB2, siERBB4 or siNTC control siRNA constructs. Bar plots depict expression relative to TOV-21G-control-siNTC cells with error bars depicting 95% confidence intervals. MRPL19 expression was used as the calibrator. One-way ANOVA (p < 0.0001) and Tukey’s Multiple Comparison tests were used to determine statistical significance. Bars not sharing a common subscript are significantly different (Tukey’s p < 0.05). (TIF) [file pone.0254205.s008.tif]

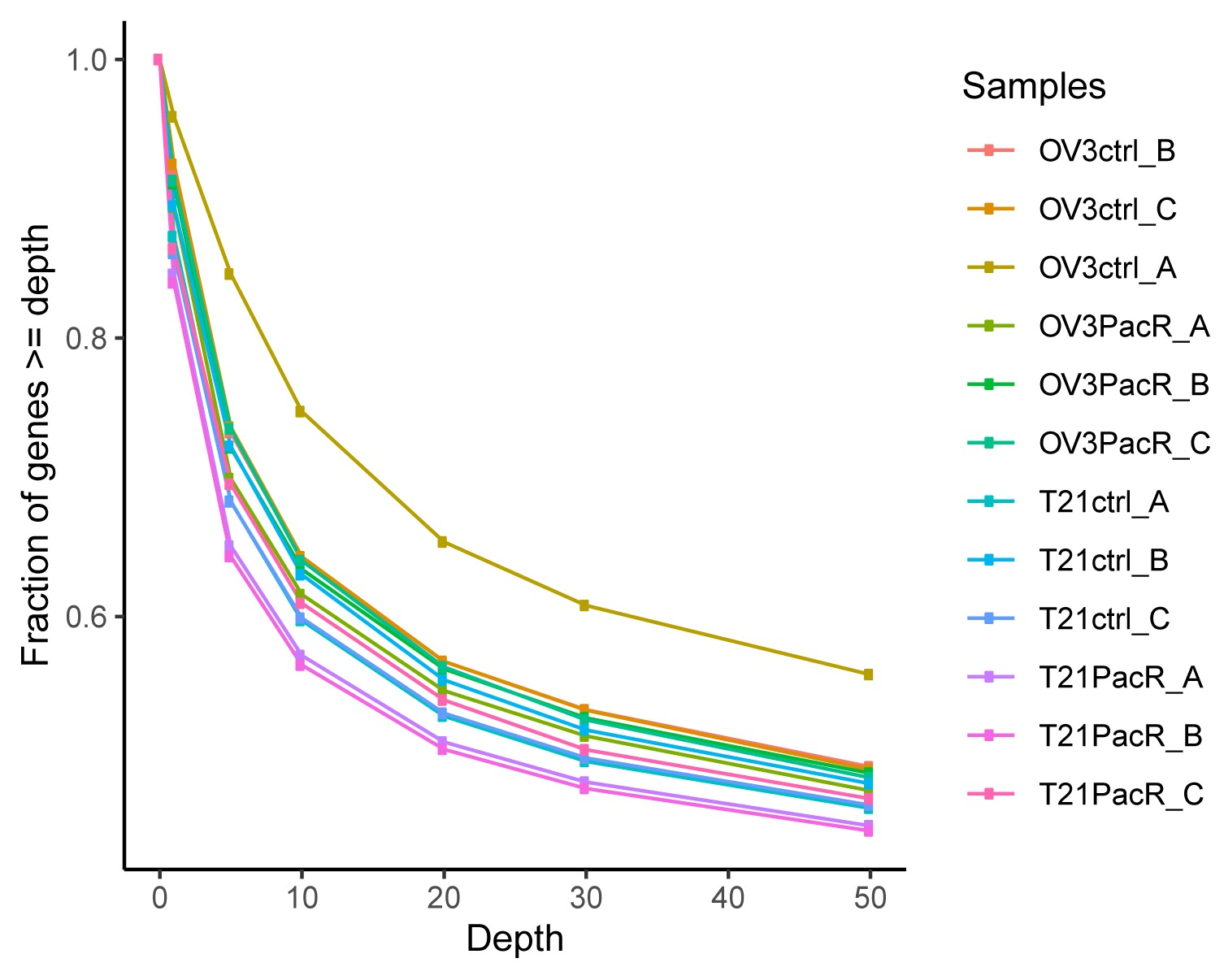

Supplement: S8 Fig — For each immortalized ovarian cancer cell line analyzed by RNA-seq, depth of coverage (x-axis) is plotted against the proportion of genes greater than or equal to a given read depth (y-axis). (TIF) [file pone.0254205.s009.tif]
